# Development and validation of nomograms to forecast overall survival and cancer-specific survival in Asian patients diagnosed with epithelial ovarian cancer

**DOI:** 10.3389/fsurg.2025.1443605

**Published:** 2025-03-25

**Authors:** Hao He, Xin Cheng, Mengna Zhao, Shimeng Wan, Shijie Yao, Hongbing Cai

**Affiliations:** ^1^ Department of Gynaecological Oncology, Zhongnan Hospital of Wuhan University, Wuhan, Hubei, China; ^2^ Department of Gynecology, Xianning Central Hospital, The First Affiliated Hospital of Hubei University of Science and Technology, Xianning, Hubei, China; ^3^ Department of Obstetrics, Lanzhou University First Affiliated Hospital, Lanzhou, Gansu, China

**Keywords:** epithelial ovarian cancer, Asian females, nomogram, SEER, overall survival, cancer-specific survival

## Abstract

**Objective:**

Asian females with ovarian cancer have different clinicopathological characteristics compared with other races. However, an effective prognostic prediction tool is lacking. The goal of our study was to develop and evaluate nomograms for estimating overall survival and cancer-specific survival in Asian patients with ovarian cancer.

**Methods:**

We extracted data from 2010 to 2018 in the Surveillance, Epidemiology, and End Results database, focusing on Asian/Pacific Islander females that had been diagnosed with epithelial ovarian cancer. To find prognostic factors, least absolute shrinkage and selection operator Cox regression and multivariate Cox regression analyses were used. Based on the outcomes, nomograms were then constructed. Numerous techniques, such as the C-index, calibration plots, decision curve analysis, and risk subgroup stratification, were used to assess the performance of the nomograms.

**Results:**

Nomograms were created to evaluate overall survival and cancer-specific survival rates over three and five years. The C-indices for overall survival and cancer-specific survival in the training cohort were 0.768 and 0.778, respectively. The C-indices for overall survival and cancer-specific survival in the validation cohort were 0.804 and 0.812, respectively. The calibration plots showed that the nomogram forecasts and actual survival results agreed. Additionally, the decision curve analysis curves indicated that the nomogram outperformed the American Joint Commission on Cancer staging system in terms of predictive accuracy.

**Conclusion:**

Nomograms and a risk classification system were created to forecast the overall survival and cancer-specific survival of Asian females with ovarian cancer. The nomograms and risk stratification system have the potential to provide valuable assistance in making future clinical decisions.

## 1 Introduction

Ovarian cancer (OC) is a highly aggressive form of gynaecological cancer, ranking as the seventh most prevalent cancer among women (1). More than 90% of all instances of OC are epithelial (EOC). It is also recognized as the most lethal form of malignancy worldwide (2). Most OC cases are distributed across East, South, and Southeast Asia, causing a heavy disease burden.

The prognosis for patients with OC is still poor despite recent increases in survival rates. Previous research has indicated that Asians may have distinct prognostic outcomes compared with other racial groups (3, 4). Nonetheless, the prognostic factors in Asian patients with EOC require further study. Many factors, such as stage, histology, and surgical methods used have been proposed to influence the prognosis of EOC (5). In addition, demographic characteristics, lymph node (LN) status, other treatments, and organ metastasis should be considered when evaluating survival outcomes.

A nomogram is a predictive tool that contains important predictors and is frequently used to evaluate risks and determine the prognosis for various cancer types. Besides, nomograms are widely used in gynecologic oncology not only for prognostic purposes but also for evaluating surgical feasibility, particularly in minimally invasive surgery (MIS) (6–8). To forecast survival rates in individuals with EOC, several prognostic models have been created. For instance, using information from the CALYPSO trial, Lee et al. (9) created a nomogram to forecast survival in patients with recurrent platinum-sensitive OC. To the best of our knowledge, no specific nomograms or prediction models have been created to predict the outcomes of Asian females with EOC.

The Surveillance, Epidemiology and End Results (SEER) database is a thorough assemblage of information on cancer from 18 American registries. Approximately 27.8% of the population is covered, and it offers useful details on people who have been diagnosed with cancer. In this study, our objective was to extract data from the SEER database, specifically on Asian females diagnosed with OC. The aim was to utilise this data to develop nomograms that can accurately forecast the prognostic outcomes of Asian females with EOC.

## 2 Materials and methods

### 2.1 Data collection

The National Cancer Institute's SEER program was used to obtain the data for this investigation. We downloaded the SEER*Stat software, version 8.3.9 from the official website (https://seer.cancer.gov/) to extract patients' data. Given that the SEER database is a public resource, it was exempt from informed consent requirements and institutional research ethics committee approval.

We chose patients who were diagnosed with EOC from 2010 to 2018 based on the International Classification of Diseases for Oncology, 3rd edition (ICD-O-3) morphology codes “8310/3, 8323/3, 8380/3, 8441/3, 8460/3, 8461/3, 8470/3, and 8480/3”. All patients had Asian/Pacific Islander (API) ethnicity. The study collected various information including age, laterality, grade, American Joint Committee on Cancer (AJCC) stage, histology, surgical procedures, chemotherapy, radiotherapy, organ metastasis, the quantity of examined LNs, LN status, vital status, and survival duration. The organs where metastasis occurred were the liver, lung, and bone.

The following conditions were the inclusion criteria for the study: (1) having EOC as their initial and primary tumour; (2) being diagnosed with EOC through histology or cytology; (3) being 18 years of age or older; (4) having complete clinicopathological characteristics, demographic information, and follow-up data. The following criteria were used for exclusion: (1) EOC not being the primary tumour; (2) lack of histological confirmation; (3) age below 18 years of age; (4) survival time less than 6 months; and (5) insufficient information available for LNs, grade, stage, surgery, chemotherapy, radiotherapy, laterality, and organ metastasis.

### 2.2 Statistical analysis

Two pieces of software, SPSS (version 26.0; IBM Corp., Armonk, NY, USA) and R (version 4.1.0; accessible at https://www.r-project.org), were used for the statistical analysis. Statistical significance was determined when *p* < 0.05.

A training cohort and a validation cohort were randomly formed from the patients with a ratio of 7:3. To compare the clinicopathological characteristics between the training and validation cohorts, the chi-square test was used. Overall survival (OS) and cancer-specific survival (CSS) were the study's primary outcomes.

We utilized the “glmnet” and “rms” packages to perform the least absolute shrinkage and selection operator (LASSO) and multivariate Cox regression analysis. LASSO regression was utilised to primarily select useful predictive elements, to avoid overfitting. Thereafter, multivariate analysis from the Cox proportional hazard regression model was utilised to identify the significant predicting variables further. Based on these results, we created nomograms to forecast OS and CSS.

To evaluate the nomograms' accuracy, we used the receiver operating characteristic (ROC) curve and the C-index, which has a range of 0.5 to 1.0. Additionally, for the purpose of assessing the consistency between the anticipated and actual 3- and 5-year OS and CSS rates, calibration curves (1,000 bootstrap resamples) were constructed. To evaluate the potential worth of the nomograms, we performed decision curve analysis (DCA). Based on the median risk score that we determined from the nomograms, we separated the entire cohort into low-risk and high-risk groups. After that, we used Kaplan–Meier analysis to compare the survival rates of the different risk subgroups.

## 3 Results

### 3.1 Baseline characteristics and survival outcomes

Overall, the SEER database yielded 1,563 patients with EOC (Table 1). All patients' ethnicity was API and the patients had a median age of 55 years. A total of 517 (33.1%) patients were younger than 50 years, 535 patients (34.2%) were aged 50–60 years, and 511 (32.7%) were over the age of 60. Most individuals were unilateral (64.1%) and had a grade of III/IV (70.5%). The most common histological type in the cohort was serous, accounting for 50.1% of cases. Endometrioid tumours accounted for 18.9% of cases, followed by clear cell tumours at 15.3%. As for the AJCC stage, most subjects were diagnosed at stage I (37.2%) and stage III (36.0%), while a lower percentage were diagnosed at stage IV (15.0%) and stage II (11.9%). Distant organ metastases were found in 84 patients (5.4%). The most common organ metastasis was to the lungs (2.9%), followed by the liver (2.6%), and bone (0.5%).

**Table 1:** Clinical and pathological features of patients in the study cohort.

Variables	Training Cohort	Validation Cohort	*P*
Total	*n* = 1,094	*n* = 469	
Age (%)			0.674
<50 years	357 (32.6)	160 (34.1)	
50–60 years	382 (34.9)	153 (32.6)	
>60 years	355 (32.4)	156 (33.3)	
Laterality (%)			0.18
Unilateral	713 (65.2)	289 (61.6)	
Bilateral	381 (34.8)	180 (38.4)	
Histology (%)			0.393
Serous	556 (50.8)	227 (48.4)	
Mucinous	90 (8.2)	49 (10.4)	
Endometroid	204 (18.6)	92 (19.6)	
Clear cell	164 (15.0)	75 (16.0)	
Mixed	80 (7.3)	26 (5.5)	
AJCC Stage (%)			0.48
I	398 (36.4)	183 (39.0)	
II	132 (12.1)	54 (11.5)	
III	391 (35.7)	171 (36.5)	
IV	173 (15.8)	61 (13.0)	
Grade (%)			0.624
I	143 (13.1)	54 (11.5)	
II	178 (16.3)	87 (18.6)	
III	432 (39.5)	180 (38.4)	
IV	341 (31.2)	148 (31.6)	
LN examined, *n* (%)			0.189
No	375 (34.3)	148 (31.6)	
1–10	304 (27.8)	120 (25.6)	
>10	415 (37.9)	201 (42.9)	
LN (%)			0.308
Not examined	912 (83.4)	381 (81.2)	
or Negative			
Positive	182 (16.6)	88 (18.8)	
Surgery (%)			0.966
Yes	1,075 (98.3)	461 (98.3)	
No	19 (1.7)	8 (1.7)	
Chemotherapy (%)			0.905
Yes	866 (79.2)	370 (78.9)	
No/Unknown	228 (20.8)	99 (21.1)	
Radiotherapy (%)			0.595
Yes	19 (1.7)	10 (2.1)	
No/Unknown	1,075 (98.3)	459 (97.9)	
Organ Metastasis (%)			0.078
Yes	66 (6.0)	18 (3.8)	
No	1,028 (94.0)	451 (96.2)	
Liver Metastasis (%)			0.162
Yes	32 (2.9)	8 (1.7)	
No	1,062 (97.1)	461 (98.3)	
Lung Metastasis (%)			0.248
Yes	35 (3.2)	10 (2.1)	
No	1,059 (96.8)	459 (97.9)	
Bone Metastasis (%)			0.939
Yes	5 (0.5)	3 (0.6)	
No			
Month	1,089 (99.5)	466 (99.4)	
(Median)	51.5	52	

Only 1.7% of patients did not undergo surgery, while 1,040 (66.5%) underwent lymph node dissection. A total of 1,236 (79.1%) patients received chemotherapy, whereas only 29 (1.9%) received radiotherapy. The two cohorts showed comparable and balanced characteristics (Table 1).

The OS rates over three and five years for all patients were 79% and 64%, respectively. The mean follow-up period was 76.7 [74.7–78.6] months. For all patients, the CSS rates over three and five years were 80% and 66.7%, respectively. The mean follow-up period was 78.9 [77.0–80.9] months. In terms of histology subtypes, serous histology had the worst 3-year and 5-year survival rate, endometrioid histology had the best 3-year and 5-year survival rate, and the other histology types had 3-year and 5-year survival rate in between. Table 2 displays the OS and CSS rates over three and five years for all patients across various clinical characteristic variables.

**Table 2:** The 3-and 5-year OS and CSS according to patient characteristics.

Variables	Overall Survival		Cancer-Specific Survival	
	3-year (%)	5-year (%)	3-year (%)	5-year (%)
Age				
<50 years	83.4	70.9	84.5	72.4
50–60 years	79.3	65.1	80.2	67.1
>60 years	74	56.9	74.4	60.3
Laterality				
Unilateral	84.4	72.6	85.9	75.9
Bilateral	69	49.8	69	50.8
Histology				
Serous	72.6	52.7	73.3	54.9
Mucinous	81.6	77.5	84.1	79.8
Endometroid	92.6	83.9	93.8	86.2
Clear cell	79.7	68.5	80	71.5
Mixed	81.6	69.7	82	71.2
AJCC Stage				
I	94	87.3	95.2	89.9
II	85.6	76.8	87.4	79.8
III	68.3	46.2	68.5	47.7
IV	62.2	40.8	62.7	44.7
Grade				
I	92.5	85.1	93.5	87.1
II	89.7	80.5	90.8	82.8
III	73.8	54.1	73.7	57.2
IV	75.5	60.3	76	61.7
LN examined, *n*				
No	70	53.7	71.4	56.7
1–10	81.2	66.3	81.9	69.4
>10	84.8	72	85.3	73.3
LN				
Not examined or Negative	81.4	68.8	82.6	71.7
Positive	66.8	42.9	67	43.5
Surgery				
Yes	79.4	64.9	80.3	67.2
No	48.1	–	45.8	–
Chemotherapy				
Yes	76.9	61.7	77.6	63.6
No/Unknown	86.7	74.6	88.6	79
Radiotherapy				
Yes	65.5	–	65.5	–
No/Unknown	79.1	64.6	80.1	67
Organ Metastasis				
Yes	65.5	51.5	66.2	51.2
No	79.7	65.1	80.5	67.4
Liver Metastasis				
Yes	67.5	–	68.4	–
No	79.2	64.6	80.1	66.9
Lung Metastasis				
Yes	64.4	43.3	63.4	45.4
No	79.3	65	80.3	67.4
Bone Metastasis				
Yes	–	–	–	–
No	79	64.5	79.9	66.9

AJCC, American Joint Committee on Cancer; LN, lymph node.

### 3.2 Independent prognostic factors for OS and CSS

**Figure 1:**
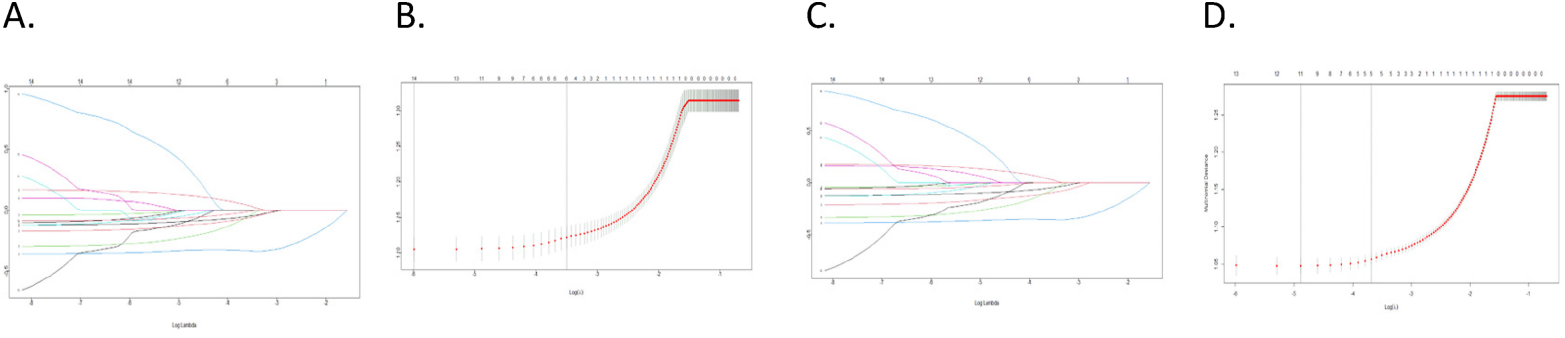
LASSO regression was utilized for variable selection; **(A,B)** LASSO regression selected six factors for OS; **(C,D)** LASSO regression selected six factors for CSS.

Overall, 14 different variables were examined. Based on findings by the LASSO regression analysis, the following elements were found to be prognostic factors for OS and CSS: grade, laterality, AJCC stage, age, LN examined, and LN status (Figure 1). According to multivariate Cox regression analysis, the AJCC stage, grade, age, LN examination, and LN status were independently and significantly related to OS, whereas the AJCC stage, grade, LN examination, and LN status were independently and strongly related to CSS (Table 3). Based on the uncovered prognostic parameters, we created nomograms to forecast the OS and CSS over three and five years.

**Table 3:** Multivariate Cox analysis of the variables based on the results of lasso regression.

Variables	OS			CSS		
	HR (95% CI)		*P*	HR (95% CI)		*P*
Laterality						
Unilateral		1			1	
Bilateral	1.129 (0.942–1.354)		0.19	1.178 (0.971–1.431)		0.097
Age, years						
<50		1			1	
50–60	1.180 (0.949–1.466)		0.136	1.181 (0.939–1.484)		0.155
>60	1.256 (1.015–1.554)		**0**.**036**	1.201 (0.956–1.507)		0.115
Grade						
I		1			1	
II	1.280 (0.799–2.050)		0.305	1.210 (0.722–2.028)		0.469
III	1.994 (1.315–3.023)		**0**.**001**	1.927 (1.227–3.028)		**0**.**004**
IV	1.707 (1.118–2.606)		**0**.**013**	1.704 (1.079–2.693)		**0**.**022**
AJCC stage						
I		1			1	
II	1.773 (1.205–2.609)		**0**.**004**	2.027 (1.311–3.134)		**0**.**001**
III	3.754 (2.765–5.097)		**<0**.**001**	4.609 (3.257–6.522)		**<0**.**001**
IV	4.100 (2.924–5.750)		**<0.001**	5.000 (3.421–7.309)		<**0.001**
LN examined (*n*)						
No		1			1	
1–10	0.696 (0.545–0.890)		**0.004**	0.674 (0.516–0.881)		**0.004**
>10	0.604 (0.469–0.778)		**<0.001**	0.592 (0.450–0.779)		**<0.001**
LN status						
Not examined or Negative		1			1	
Positive	1.471 (1.138–1.902)		**0.003**	1.545 (1.175–2.031)		**0.002**

AJCC, American Joint Committee on Cancer; LN, lymph node; OS, overall survival; CSS, cancer-specific survival; HR, Hazard Ratio; CI, confidence interval.

Bold values mean statistically significant.

**Figure 2:**
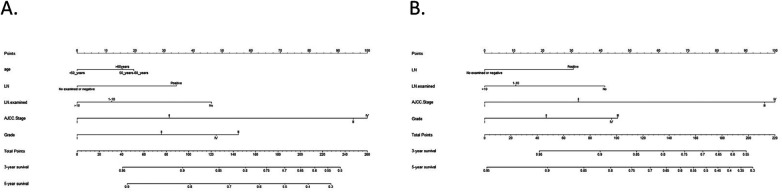
Nomograms for OS and CSS. **(A)** Nomogram for predicting 3- and 5-year OS; **(B)** Nomogram for predicting 3- and 5-year CSS.

In the training cohort, the nomograms achieved a C-index of 0.768 for OS and 0.778 for CSS. The C-indices for OS and CSS in the validation cohort were 0.804 and 0.812, respectively. To evaluate the nomogram's capacity for discrimination, we used ROC curves (Figure 2).

**Figure 3:**
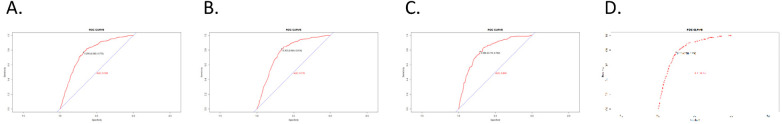
ROC curves. **(A)** OS nomogram ROC curve for training cohort; **(B)** CSS nomogram ROC curve for training cohort; **(C)** OS nomogram ROC curve for validation cohort; **(D)** CSS nomogram ROC curve for validation cohort.

**Figure 4:**
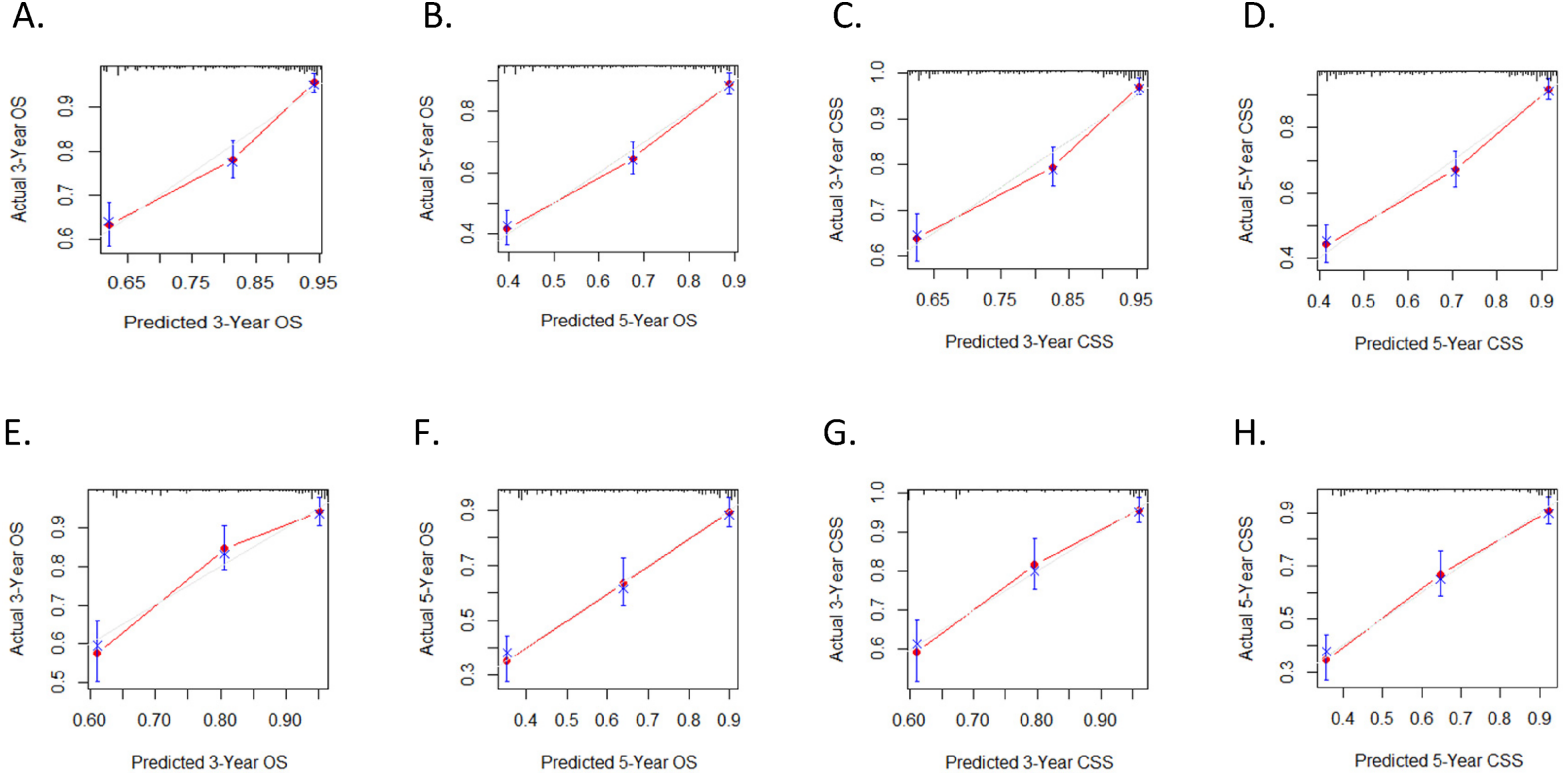
Calibration plots. (**A,B)** 3-year and 5-year OS nomogram calibration plots for training cohort; (**C,D)** 3-year and 5-year CSS nomogram calibration plots for training cohort; **(E,F)** 3-year and 5-year OS nomogram calibration plots for validation cohort; **(G,H)** 3-year and 5-year CSS nomogram calibration plots for validation cohort.

**Figure 5:**
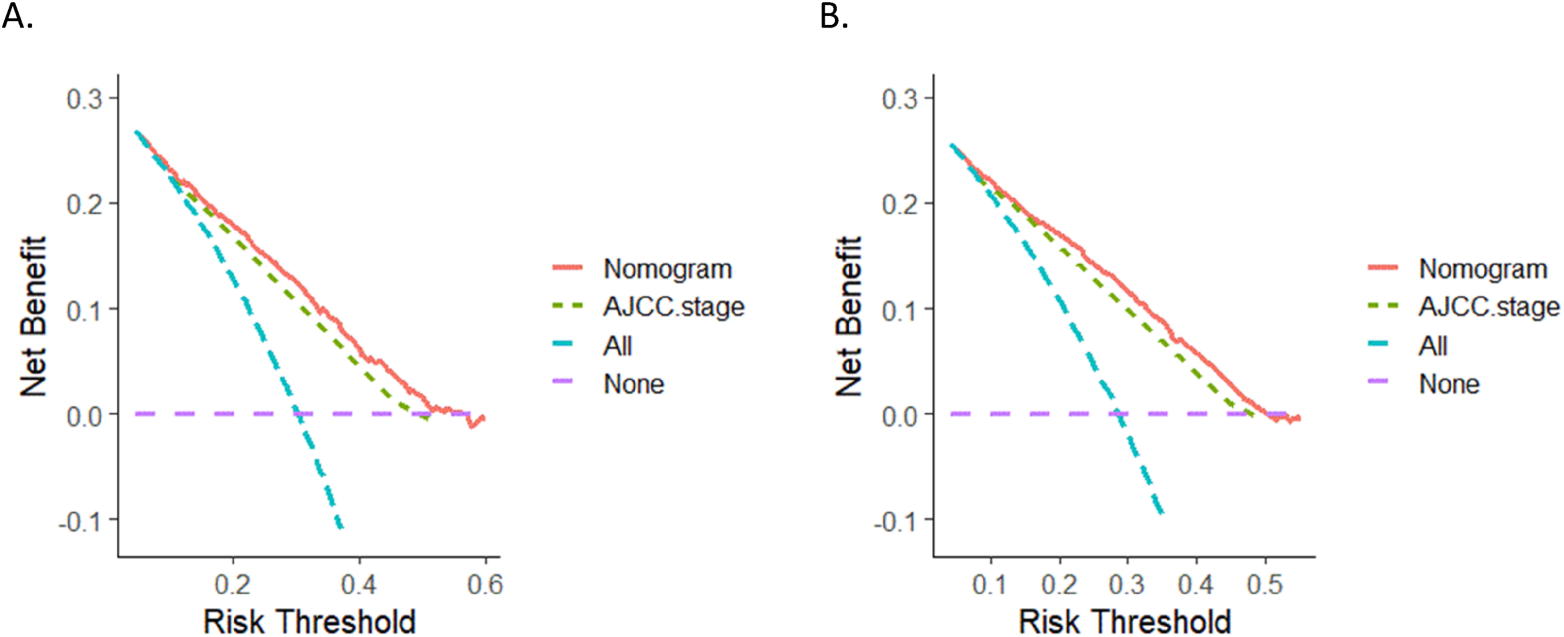
DCA curves. **(A)** DCA curve of the nomogram and AJCC stage for OS; **(B)** DCA curves of the nomogram and AJCC stage for CSS. DCA, decision curve analysis.

The calibration curves for three and five years of OS and CSS demonstrated an excellent concordance between the predictions of the nomogram and the observed survival rates in both the training group and the validation cohort (Figure 3). The DCA curves revealed that the nomogram models outperformed the AJCC staging system and offered good forecasts (Figures 4, 5).

### 3.3 Risk stratification of Asian females with OC

**Figure 6:**
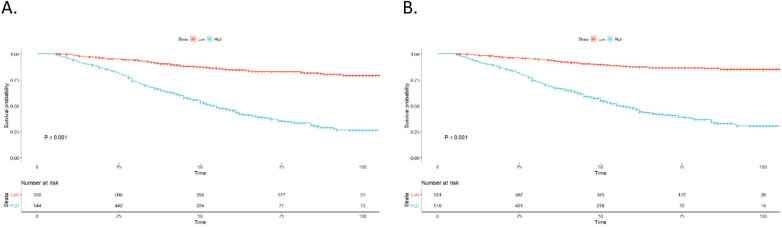
Kaplan–Meier curves. **(A)** OS for patients stratified by the risk stratification system; **(B)** CSS for patients stratified by the risk stratification system.

The nomogram assigned a risk score to each variable. The sum of the scores for all variables was used to calculate the overall score for each patient. Subsequently, based on the median risk score, the cohort was split into two subgroups: low-risk and high-risk. The Kaplan–Meier survival curves for these subgroups is illustrated in Figure 6. OS and CSS were significantly different between the low-risk and high-risk groups (*p* < 0.001 and *p* < 0.001, respectively). Overall, these findings demonstrate the excellent ability of our nomogram to perform risk stratification.

## 4 Discussion

Asian patients with OC may exhibit distinct characteristics when compared to individuals of other races. In an NRG Oncology/GOG Ancillary study involving 7,914 patients, it was reported that Asian patients with OC tended to be younger and had better performance status. Additionally, clear cell and mucinous tumour subtypes were more prevalent, and they were more likely to be in the early stages of illness (3). In ovarian carcinogenesis, type II carcinomas (high-grade, clinically aggressive tumours) are more frequent in Europe and the USA than in Asia (10). In addition, the incidence and 2-year relative survival of Asian patients differ from those of other races (11).

In this study, we analysed the risk factors directly related to EOC among Asian patients using data from the SEER database. We developed nomograms according to the identified independent prognostic factors to predict the OS and CSS rates over three and five years. To evaluate the calibration and discrimination of the nomograms in both the training and validation cohorts, we used C-indices, calibration curves, and DCA curves. The findings suggested favourable consequences, demonstrating excellent performance of the nomograms. Furthermore, we successfully developed a risk stratification system utilising the risk scores generated by the nomograms.

Five independent OS prognostic variables were found in this study: age, tumour grade, AJCC on Cancer stage, LN examined, and LN status. These same factors (except age) also significantly impacted CSS. In our study, we observed a negative correlation between age and survival outcomes, indicating that older patients tended to have worse survival. Older patients often experience decreased immune responses, which may contribute to their poorer survival (12). A study conducted by Fuh et al. (3) reported that Asians are more likely than Caucasians to present with stage I illness and clear cell malignancies. In this study, Asian patients were more likely to present with both stage I and stage III illnesses. Furthermore, serous was the most common histological subtype with poor prognosis, followed by endometrioid and clear cell.

Although lymphadenectomy is considered an important part of the National Comprehensive Cancer Network **(**NCCN) treatment guidelines (13), the prognostic value of lymphadenectomy remains controversial. According to research by Paik et al. (14), patients with advanced OC who underwent a primary debulking surgery with lymphadenectomy had better survival rates, particularly in terms of survival after recurrence. A multi-institutional retrospective study found that lymphadenectomy performed for advanced, high-grade serous OC with R0 status after primary debulking surgery was associated with improved oncologic outcomes (15). In early-stage OC, suboptimal staging is an indicator of quality of care, and tumor biology, as defined by histological subtypes such as low-grade endometrioid and mucinous types, independently affects disease-free survival (DFS) (16). Furthermore, lymphadenectomy may increase OS in patients with both early-stage and advanced OC, according to two meta-analyses (17, 18). Additionally, a multicenter retrospective study by Bizzarri et al. found that staging lymphadenectomy in patients with grade 2 endometrioid ovarian carcinoma is associated with improved DFS and OS. This supports the findings in the nomogram of our study (19). Contrary to the findings of those studies, numerous retrospective investigations have demonstrated that lymphadenectomy does not significantly affect the mortality results of patients with both early-stage and advanced EOC (20, 21). The prognostic relevance of lymphadenectomy varies substantially between early and advanced stages. In a randomised trial, systemic lymphadenectomy was not found to increase OS or progression-free survival (PFS) in patients with advanced OC (22). Wang et al. found that for patients with early-stage ovarian cancer, lymphadenectomy does not significantly improve progression-free or overall survival rates (23). However, Ronsini et al. found that for advanced stage, lymphadenectomy is protective and reduces the risk of death (24). These findings suggest that lymphadenectomy should be carefully considered for different stages of ovarian cancer.

According to our research, patients who underwent surgery to remove more than ten LNs had much better prognosis and survival rates. In our study, we identified the LN status as a significant prognostic factor and the patients' prognosis was discovered to be related to their LN status. The underlying mechanisms of systemic lymphadenectomy should be elucidated in future studies.

Numerous studies have consistently demonstrated the superior performance of the nomogram model in prognosticating OC compared to traditional staging systems. The nomogram has been proposed as a promising tool for prognostic assessment for this disease. For instance, Chen et al. (25) developed and validated nomograms for predicting OS and CSS in patients diagnosed with ovarian clear cell carcinoma. Their nomograms obtained C-indices of 0.802 [95% CI: 0.773–0.831] for OS and 0.802 [95% CI: 0.769–0.835] for CSS, indicating strong predictive accuracy. Liu et al. (26) created an externally validated nomogram to forecast OS in patients diagnosed with serous ovarian carcinoma, who underwent satisfactory surgery and chemotherapy. Their nomogram obtained a C-index of 0.689 [95% CI: 0.677–0.701], indicating reasonable predictive accuracy. Zhang and Zhu (27) created an internally validated nomogram to forecast early cancer-specific mortality following surgery in patients diagnosed with EOC. The nomogram demonstrated a robust predictive capability for cancer- specific early death, as evidenced by a high C-index of 0.787 [95% CI: 0.772–0.794]. All these nomograms demonstrated superior discriminatory capacity compared to traditional staging systems.

In our work, we carried out a thorough validation of the precision of our nomograms, utilising various metrics, including C-indices, ROC curves, calibration curves, and DCA. Both the training and validation cohorts underwent these evaluations to guarantee the accuracy of our nomograms. The C-indices for OS and CSS were found to be 0.768 and 0.778, respectively, indicating good discriminative ability. The ROC curves further supported the favourable performance of the nomograms in predicting survival outcomes. Additionally, the DCA curves and the calibration curves confirmed the optimal clinical applicability and calibration of the nomograms, respectively, highlighting their accuracy and usefulness in prognostic prediction.

Our study possesses several notable strengths. For example, we utilised a large sample size from the SEER database, which provided high-quality data for constructing and validating our nomograms. To the best of our knowledge, no specific nomograms of Asian patients with OC using the SEER database existed before we constructed our nomograms. The fact that the nomograms we created outperformed the AJCC 7th edition staging system in terms of predictive ability is another significant benefit of our study. This performance was confirmed through DCA. DCA is a valuable tool in assessing the clinical utility of prediction models. It goes beyond traditional performance metrics by incorporating the clinical judgement of the potential benefits and harms associated with the use of a specific model (28). In addition to its predictive value, the nomogram model has the advantage of enabling risk stratification of patients to facilitate different treatment strategies.

Nevertheless, it is important to acknowledge that the current study includes several limitations. First, this study made use of retrospective data, which may inevitably introduce selection bias. Second, detailed information, such as specific chemotherapy regimens and cycles, ascitic fluid, genetic results such as BRCA mutations and homologous recombination deficiency, family history of OC, location of positive LNs, and recurrence, were unavailable. Third, The influence of confounding factors such as socioeconomic factors and healthcare factors on prognosis could not be fully evaluated.The impact of these factors needs to be further evaluated in prospective studies. Fourthly, it is important to acknowledge that the nomogram model developed in this study only underwent internal validation using the available data.External validation using independent cohorts and prospective studies is essential to confirm the performance and generalisability of the nomogram model. Finally, the prognostic nomogram was developed based on both early- and advanced-stage cases. This is a significant limitation. As we discussed above, the prognostic relevance of lymphadenectomy varies substantially between these two stages. More studies on lymphadenectomy for ovarian cancer at different stages are needed in the future.

## 5 Conclusion

We utilised a large-scale database in our study, which allowed us to construct nomograms that demonstrated favourable predictive capacity for assessing the OS and CSS rates over three and five years in Asian females with OC. We also created a risk stratification system utilising risk scores generated by the nomograms. This risk stratification system categorises patients into different risk groups. For high-risk groups, therapeutic measures could be actively taken in clinical work, such as sentinel lymph node dissection to clarify the status of lymph nodes, or more lymph node dissection to improve the prognosis of these patients.In the future we hope to integrate nomograms into clinical workflows to make more precise prognostic guidance in clinical practice.

## Data Availability

The raw data supporting the conclusions of this article will be made available by the authors, without undue reservation.
